# Language Contact Within the Speaker: Phonetic Variation and Crosslinguistic Influence

**DOI:** 10.1177/00238309231182592

**Published:** 2023-07-31

**Authors:** Khia A. Johnson, Molly Babel

**Affiliations:** Department of Linguistics, The University of British Columbia, Canada

**Keywords:** Speech production, bilingualism, pronunciation variation, crosslinguistic influence, spontaneous speech corpus

## Abstract

A recent model of sound change posits that the direction of change is determined, at least in part, by the distribution of variation within speech communities. We explore this model in the context of bilingual speech, asking whether the less variable language constrains phonetic variation in the more variable language, using a corpus of spontaneous speech from early Cantonese–English bilinguals. As predicted, given the phonetic distributions of stop obstruents in Cantonese compared with English, intervocalic English /b d g/ were produced with less voicing for Cantonese–English bilinguals and word-final English /t k/ were more likely to be unreleased compared with spontaneous speech from two monolingual English control corpora. Whereas voicing initial obstruents can be gradient in Cantonese, the release of final obstruents is prohibited. Neither Cantonese–English bilingual initial voicing nor word-final stop release patterns were significantly impacted by language mode. These results provide evidence that the phonetic variation in crosslinguistically linked categories in bilingual speech is shaped by the distribution of phonetic variation within each language, thus suggesting a mechanistic account for why some segments are more susceptible to cross-language influence than others.

## 1 Introduction

Phonetic variation in bilingual speech stems from multiple sources. Crosslinguistic influence is a major source of variation, as the dynamics of parallel activation and inhibition of competing linguistic units can influence pronunciation patterns. While these pronunciation patterns are typically discussed as *interference* in speech production (Grosjean, 2011; Simonet & Amengual, 2019), the information is beneficial to listeners, as bilinguals use this phonetic variation in pronunciation patterns to efficiently predict and process an upcoming code-switch (Fricke et al., 2016; Shen et al., 2020). Crosslinguistic influence can also be a stable signature of a multilingual or nonnative accent, and is termed as *transfer* in such cases (Grosjean, 2011). In these cases, the resulting phonetic variation reflects a more codified or systematic linguistic pattern shaped by the bidirectional nature of influence in bilinguals’ linguistic systems, as opposed to the arguably more localized and temporary effects of parallel activation. As a pattern becomes more systematic, it can be challenging to distinguish more ephemeral variation from a dialect. These points likely form a continuum, given that new language varieties develop out of language contact (Schneider, 2003).

We are interested in *how* early bilinguals’ language systems influence one another—specifically, how crosslinguistic influence at the level of the speech community may lead to the development of novel language varieties. Certainly, on the community level, contact-induced language change is a well-studied entity (see, for example, the seminal book by Thomason & Kaufman, 1988). Moreover, decades of research on second-language (L2) acquisition has established that bilingual speakers make phonological connections between their languages, presumably providing the cognitive infrastructure that supports mutual influence (e.g., Best & Tyler, 2007; Chang, 2015; Flege, 1995; Flege & Bohn, 2021). In this body of literature, the term “phonological” tends to be used in a psycholinguistic manner, rather than a theoretical phonological one. That is, while some level of abstraction and structural organization is needed to explain phenomena such as crosslinguistic influence, it does not necessarily map on to a framework in phonological theory (see Chang, 2015). This parallels Samuel’s (2020) warning that linguistic and psycholinguistic categories should not be conflated with one another in perception, and is further reflected by the revised Speech Learning Model’s (SLM’s) emphasis on phonetic and surface-level behavior at the individual level, rather than on a phonological space shared across languages (Flege & Bohn, 2021). The explicit connection of psycholinguistically informed mutual influence on community patterns and language change has also been made (Tse, 2019; Van Coetsem, 2000; Winford, 2007). Yao and Chang (2016) recently presented evidence that language contact within the bilingual’s spheres of knowledge guides sound change.

In this paper—following Yao and Chang’s lead—we bring together methods from the study of crosslinguistic influence and concepts from sound change to examine Cantonese influence on English in spontaneous speech from early Cantonese–English bilinguals, using the corpus described in Johnson et al. (2020). For the purposes of the current study, the primary delineator between the sound change and crosslinguistic influence literatures is whether language contact occurs between groups of people (e.g., as in the agent-based model by Harrington et al., 2018) or within the linguistic system of a bilingual (for a recent review, see Fricke et al., 2019). Given this, we ask, how do the sound systems *within an individual* accommodate each other given mutual crosslinguistic influence? Our goal is to become more specific about the nature of the influence by adopting frameworks from sound change (Harrington et al., 2018; Harrington & Schiel, 2017).

### 1.1 Bilingualism and mutual influence

A large body of psycholinguistic research consistently finds evidence of mutual influence in bilingual speech production at various levels of the linguistic structure (Kroll & Navarro-Torres, 2018). As noted above, mutual influence can take the form of interference, which is the result of both languages being simultaneously activated during speech production. Interference has been found in cognate production (Amengual, 2012), in language-switching paradigms (Goldrick et al., 2014), by inducing a bilingual mode through the experimental context (Simonet & Amengual, 2019), and in corpus work examining code-switching behavior (Fricke et al., 2016). Clear evidence of transfer is harder to come by, in part because there are challenges with ensuring that bilinguals operate in a strictly monolingual mode, but also because the same linguistic items can be simultaneously affected by transfer and interference (Grosjean, 2011).

The focus in this body of work is on the degree (i.e., is it quantifiable and perceptible?; e.g., Sancier & Fowler, 1997) and direction of crosslinguistic influence (i.e., is it the dominant language that influences the non-dominant language?; e.g., R. K.-Y. Tsui et al., 2019). Varying degrees of phonetic convergence across linguistic systems have been found for vowels (Baker & Trofimovich, 2005; Simonet, 2011; Simonet & Amengual, 2019), /l/ (Barlow, 2014) and stops (Antoniou et al., 2010, 2011; Bullock & Toribio, 2009; Flege, 1987; Fricke et al., 2016; Goldrick et al., 2014; Y. Kang et al., 2016; Major, 1992; Olson, 2016; Sancier & Fowler, 1997; Sundara et al., 2006). There is also ample evidence indicating that bilingual speakers can maintain contrasts (e.g., Casillas, 2021; Guion, 2003; K.-H. Kang & Guion, 2006; MacLeod & Stoel-Gammon, 2005, 2009; MacLeod et al., 2009; Sundara et al., 2006). This body of literature varies, of course, in terms of the languages studied, the dominance and nature of the bilinguals, and the paradigms exploited. For example, in a single-word language-switch paradigm with Cantonese–English bilinguals, R. K.-Y. Tsui et al. (2019) found that the voice onset time (VOT) patterns of the dominant language carried over to influence the production of stops in the non-dominant language on switch trials for both self-reported Cantonese-dominant and English-dominant bilinguals. Self-reported balanced bilinguals showed no effects of language switching. Chang (2012), however, finds that the learning of a new language (L2 Korean) affects pronunciation patterns of the first—or native—language (L1 English), which was the clear dominant language in the population under study.

While factors like language dominance and mode clearly impact bilingual speech (Fricke et al., 2019; R. K.-Y. Tsui et al., 2019), they do not fully account for why some segments are more susceptible to influence than others (e.g., Bullock & Toribio, 2009; Olson, 2016), or why simultaneous bilinguals sometimes exhibit asymmetrical influence in speech production (e.g., Lein et al., 2016; Sundara et al., 2006). The notion of some segments being more susceptible is best illustrated with an example. Some researchers speculate that initial English long-lag stops allow for a wider range of acceptable VOT values than initial Spanish short-lag stops, and argue that the typical direction of influence from Spanish to English could be accounted for—in part—by this asymmetry (Bullock & Toribio, 2009; Olson, 2016). A similar observation can be made with early French–English bilinguals, who are substantially more likely to produce lead voicing for the English voiced stop series compared with monolinguals (Sundara et al., 2006). Lead voicing for initial “voiced” stops in North American English varieties is often absent, though not always. French, on the contrary, has consistent lead voicing for the equivalent sounds. For bilinguals, the more variable language—English—appears to be influenced by the more regular lead voicing found in French. This influence is asymmetrical, as the same group of early bilinguals continue to produce monolingual-like lead voicing in their French (Sundara et al., 2006). In this paper, we argue that asymmetries such as these may arise from crosslinguistic differences in the shape of category variability. We make this connection by borrowing concepts from the sound change literature.

While much of the literature on mutual influence summarized earlier suggests that there is a binary choice between convergence or maintenance, categories can also actively diverge (or dissimilate) from one another. A variety of different studies document divergence in bilingual phonetic systems (e.g., Bullock & Toribio, 2009; Flege, 1987; Flege & Eefting, 1987; Guion, 2003; Polinsky, 2018), though in some cases the distinction between divergence and maintaining contrast may be fuzzy. Early bilinguals show evidence of reorganizing their sound systems to maintain phonological contrasts across their languages. This occasionally results in phonetic distributions that split the difference between monolingual norms in either language, maintain distinctions between close contrasts in their languages, or reflect a phonetic distribution of a phonological contrast shifted somewhat in phonetic space. Many of these processes could be characterized as a kind of divergence. Guion (2003) describes this kind of scenario as the result of balancing inventory economy and phonetic dispersion in the case of the Quichua–Spanish bilingual vowel inventory. Divergent phonetic behavior can be dynamic, where the degree of similarity across languages is adjusted by speakers for intentional effect. An example of this is presented in Bullock and Toribio (2009), where Spanish teachers, who were late Spanish learners, both shorten Spanish short-lag stops and lengthen English long-lag stops when they are in close proximity to one another in elicited laboratory speech. Bullock and Toribio (2009) argue that this is a part of bilinguals’ rich repertoire of forms that can be deployed for any number of sociolinguistic reasons. Essentially, VOT in this case is an example of a dimension that a speaker can intentionally alter at their discretion, as a means of showcasing meta-linguistic awareness and making the languages more divergent. In some ways, the example of speakers’ strategically controlling their VOT to index meta-linguistic awareness underscores the importance of using conversational, spontaneous speech as the way of characterizing an individual’s linguistic behavior, as spontaneous speech mitigates the role that laboratory-induced performative speaking styles play in shaping our understanding of speech processes in general.

### 1.2 Connecting models of sound change and bilingualism

Whether characterizing the consequences of language contact as an indicator of language loss or the development of a new dialect, there has been relatively limited discussion of the underlying cognitive and psycholinguistic mechanisms that goad the linguistic transfer process (Winford, 2007). Extant models like the one described in Van Coetsem (2000) place the motivation for transfer on the dominant language, or are designed around accounting for categorical language behavior at the morpheme level (Myers-Scotton & Jake, 2017). Prominent L2 acquisition models such as the revised SLM (SLM-r; Flege & Bohn, 2021)—a further development of the seminal SLM (Flege, 1995)—are designed to account for the ways in which bilingual phonetic systems (re)organize over the lifespan, as L1 and L2 inputs waxe and wane. The SLM-r focuses on individual differences. In the SLM-r, the distributional precision of L1 categories is a core factor in accounting for whether an individual forms composite distributions of “linked” categories across their languages or keeps distinct categories for each language. Distributional precision is viewed in the SLM-r as an individual-specific factor that is potentially related to auditory acuity. The prediction within the SLM-r literature is that individuals with less precise categories will be more likely to have composite phonetic categories that exhibit strong(er) mutual influence. This mechanism was inspired by a series of experiments on vowel perception and production by Kartushina and colleagues, who find that individuals with compact L1 vowel categories tend to also have compact L2 categories, even following initial L2 exposure in the laboratory (Kartushina et al., 2015). Emphasizing the role this plays in the domain of individual differences, the SLM-r specifically states that L1 category precision is not shaped by language-specific phonetic factors, but is rather an exclusively endogenous factor determined by an individual’s auditory acuity.

As the SLM-r is intended to account for L2 acquisition, the category precision mechanism for discrete or combined categories does not necessarily fit in with what is known about individuals whose bilingualism arises from infancy and early childhood. Infant bilinguals often have imprecise phonetic categories and high auditory acuity (Werker & Byers-Heinlein, 2008), which does not neatly line up with the presumed relationship between category precision and auditory acuity for adults in SLM-r (Flege & Bohn, 2021). Such early bilinguals are well-equipped to establish similar, yet distinct phonetic categories, which presumably last into adulthood (e.g., Guion, 2003; MacLeod et al., 2009; Sundara et al., 2006). What accounts for mutual influence in this population of bilinguals? The answer to this question would almost certainly shed light on the nature of linked categories in a multilingual system. And crucially, how might that influence collectively shape a language variety under contact? These questions guide our present inquiry.

Contemporary within-language sound change research provides a mechanism—distinct from category precision—to account for why a change goes in a particular direction. Harrington and Schiel (2017) and Harrington et al. (2018) argue that when different versions of a category (e.g., within-category phonetic variation for older and younger speakers) come into contact with each other, sound change follows when one group’s distribution falls in the path of the other. That is, the skewness and direction of that skew are paramount, and not simply the variance or compactness of the distribution (cf., Kartushina et al., 2015; Kartushina & Frauenfelder, 2014; Kartushina, Frauenfelder, & Golestani, 2016; Kartushina, Hervais-Adelman, et al., 2016). To be specific, let us say we have two language models, A and B, which realize a category differently. If the direction of the variation is asymmetric such that A’s distribution is skewed toward B more than B’s toward A, then A will shift in the direction of B. Harrington and Schiel (2017) test this model with /u/-fronting data from older and younger speakers of British English, demonstrating that the change-in-progress dynamics of /u/-fronting can be accounted for within a cognitive model that is sensitive to distributional information without the need for social motivations to advance the change. Harrington and colleagues (Harrington et al., 2018; Harrington & Schiel, 2017) argue that the structure of variability can (at least partially) explain why a sound change occurs, and why it occurs in a particular direction, placing more explanatory power in the answer(s) to the actuation problem (Chen & Wang, 1975; Weinreich et al., 1968).

We extend this concept to the phonetic variation underlying systems of sound categories across languages as follows. The literature on crosslinguistic phonetic influence has generally focused on the role of language dominance or mode as a means of accounting for which language undergoes more change (i.e., convergence). The malleability of segments can be modeled in a framework such as the SLM-r (Flege & Bohn, 2021), but for cross-language sound linkages, the SLM-r does not necessarily account for asymmetries in the direction of influence in early bilinguals. The explanatory mechanism explored here is that—following the age-based variation exploited in Harrington and Schiel (2017)—the nature of the segment variability in each of the respective languages matters for linked segments across languages within a bilingual, and that some segments are more susceptible to variation than others (as observed in Bullock & Toribio, 2009; Olson, 2016; Sundara et al., 2006). We posit that this parallels the sound change observations, and that the structure of phonetic variation is key for understanding what happens to linked sounds, whether the comparison is made in the distributions between groups or in the distributions between languages within a bilingual’s mind.

Harrington and colleagues’ framework hinges on perception. The perception–production feedback loop within a bilingual is likely more complicated than that of a monolingual, as bilinguals can make connections at the sub-phonemic (e.g., Barlow, 2014), phonemic (e.g., Sundara et al., 2006), lemma (e.g., Linck et al., 2009), and social levels (e.g., Szakay et al., 2016). For example, Yao and Chang (2016) provide evidence for a reversal of the merger of /e/ and /ɛ/ in Shanghainese, which they argue was necessarily supported by perceptual connections at the lexical–phonetic level, and not only at the allophonic level, as the unmerging was not a wholesale shift of the sounds in every environment, but tied to particular lexical sets. The crosslinguistic connections at the word level facilitated and spurred this particular unmerging. In bilinguals, the perceptual links between segments may not actually be at a sensory-based perceptual level—at least not exclusively—but rather comprise part of the cognitive organization of linguistic structure (e.g., see discussion of abstraction and linkage in Chang, 2015). The voiced/unaspirated stop series in English and Cantonese, for example, are linked by their surface-level similarities in onset position, where they can both surface as short-lag stops but participate in different phonological patterns within each language, constrained by language-specific phonotactics (Bauer & Benedict, 2011). In a separate example, /p t k/ can occur in English in the initial and final positions, as well as in consonant clusters, whereas the equivalent long-lag Cantonese stop series /p^h^ t^h^ k^h^/ is licit syllable-initially in Cantonese and does not occur in consonant clusters. Equivalent is intended here in the sense of Chang’s (2015) relative phonetics, as the two series occupy the long VOT niche in each language, regardless of how they might be theoretically specified. In addition, Cantonese stops occur in the final position, though the underlying form is ambiguous as all final stops are unreleased at the surface level. What is crucial here, however, is that the locus of these connections is within the bilingual and the consequence of the distributional dynamics à la Harrington and colleagues’ model may come to fruition.

### 1.3 Cantonese and Cantonese–English bilingualism

In this study, we consider spontaneous speech from a corpus of early Cantonese–English bilinguals—the corpus is described in more detail below, and in Johnson et al. (2020). Here we provide a brief summary of Cantonese and English in Hong Kong and Canada, as these speech communities are most connected to the bilinguals studied here.

Cantonese is a Yue language with over 80,000,000 speakers; the largest Cantonese-speaking population is centered in the Guangdong province in southern China (Eberhard et al., 2020). A snapshot of the obstruent contrasts in word-initial position receiving our focus in this paper is presented in Table 1. The International Phonetic Alphabet (IPA) symbols used to denote these obstruents are taken from the literature on English and Cantonese (Bauer & Benedict, 2011; Zee, 1999), though there are relevant deviations from these symbolic choices for Cantonese (Matthews & Yip, 2013). The Cantonese word-initial series denoted here by /p t k/ is described as voiceless unaspirated, and Bauer and Benedict (2011) explicitly connect this series to the phonologically voiced stop series in English: /b d g/. They highlight that while the English voiced stops are variably voiced, the Cantonese equivalent series is never voiced. Matthews and Yip (2013) opt to use the symbols /b d g/ to describe the voiceless unaspirated series in Cantonese. Together, this suggests that the Cantonese voiceless unaspirated word-initial stop series and English’s voiced stop series may be linked for bilingual speakers. Such linkages are more often informed by abstract, relative, phonetic similarity than raw acoustic-auditory similarity (see discussion in Chang, 2015), though both abstract and physical similarity seem to align for Cantonese and English in this case. The voiceless aspirated series /p^h^ t^h^ k^h^/ in Cantonese and the voiceless series in English /p t k/ align in a more straightforward manner, given that in both languages these sounds are described as voiceless and aspirated (Matthews and Yip, 2013; Mielke & Nielsen, 2018), and occupy similar relative positions in their respective inventories, regardless of the phonological features ascribed to the series in any given theoretical framework. Cantonese phonology permits stops in syllable-final position and these are described categorically as unreleased in grammars (Bauer & Benedict, 2011; Matthews & Yip, 2013). A caveat for the coronal series is that the Cantonese coronals are typically described as more dental (Bauer & Benedict, 2011), whereas English is more alveolar. Cantonese also has a labialized velar series that does not have a phonological match in English and is therefore neither discussed nor addressed further in this work.

**Table 1.** Stop Places of Articulation in North American English and Cantonese That Are Relevant for a Cross-Inventory Comparison Represented With IPA Symbols Typical in the Literature.

	Labial	Coronal	Dorsal
North American English	b p	d t	g k
Cantonese	p p^h^	t t^h^	k k^h^

Multilingual competence is characteristic of Cantonese speakers within and outside the linguistic homeland of Cantonese. L1 speakers of Cantonese also often speak Mandarin (Putonghua) and English, particularly those in Hong Kong, which was colonized and occupied by the British for nearly 100 years. Owing in part to its colonial history, English is the lingua franca in Hong Kong. Hong Kong English is typically characterized as an emergent variety (Schneider, 2003; Sung, 2015), and while there is no consensus on whether Hong Kong English can yet be labeled as a codified variety, scholars assert that there is an identifiable native Hong Kong English accent (Hung, 2000; Sung, 2015). The literature on Hong Kong English consistently describes a contracted phoneme inventory compared with Received Pronunciation (RP), the British English variety to which it is most often compared (Deterding et al., 2008; Sewell & Chan, 2010; Sung, 2015). While it is frequently noted that English voiced fricatives are often devoiced in Hong Kong English (Bolton & Kwok, 1990; Hung, 2000; Leung & Brice, 2012; Sewell & Chan, 2010; Sung, 2015), there is less discussion of the stop series. Going back several decades, Bolton and Kwok (1990) note that word-final stop consonants are sometimes deleted, but also include an example of postvocalic final /t/. In the context of final consonant clusters in Hong Kong English, Deterding et al. (2008) report that final /t/ is deleted 47% of the time and /k/ 59% of the time. Comparing Hong Kong English with RP, Hung (2000) uses word list data from a small sample of university students (n=15) to characterize various aspects of Hong Kong English phonology. He describes word-initial English voiced stops as not being truly voiced in Hong Kong English, noting that the voiced stop series is distinguished from the voiceless stop series in terms of the VOT lag, as is the case in many varieties of English. Hung (2000) also characterizes Hong Kong English voiced and voiceless stops in intervocalic position as being differentiated by VOT lag, as opposed to the voicing contrast typical of this environment across many English varieties. For stops in word-final position, he describes Hong Kong English voiced stops as being unreleased and voiceless stops including an aspirated release in his word list data. This, he describes, is a systematic phonology with influence from Cantonese, but not one that can be attributed to wholesale application of Cantonese phonology into an English system. Setter et al. (2010) describe Hong Kong English as heterogeneous, though it is worth highlighting that all languages exhibit variation (Laver & Trudgill, 1979).

The Cantonese-speaking population in Canada is a diaspora that has extended roots (Yu, 2013), but the evidence about whether the English of Cantonese–English bilinguals in Canada carries any unique signatures comparable to Hong Kong English is somewhat mixed. Controlled laboratory-elicited read sentences suggest pronunciation differences, if extant, are not perceptible to listeners (Babel & Russell, 2015), but more natural semi-spontaneous speech from early Cantonese–English bilinguals is categorized as associated with Chinese identity at levels well above chance (P. Wong & Babel, 2017). Hoffman and Walker (2010) examined the speech patterns of ethnically Chinese individuals in Toronto, who were either first-generation Cantonese-dominant speakers (n=10) or second- or third-generation English-dominant speakers from historically Cantonese-speaking families (n=23). Speech data were collected via interviews conducted by individuals who were “themselves members of the relevant communities” (Hoffman and Walker, 2010, p. 45). T/d deletion was examined in word-final position using a subsample of their speakers such that they had 50–100 tokens per talker in select word-final consonant clusters. T/d deletion was categorically coded as deleted or present. Both first- and second-/third-generation ethnically Chinese individuals showed more deletion than the control white monolingual speakers descended from British Isles populations (n=20). Hoffman & Walker (2010) interpret their results as suggesting language transfer in the speech of first-generation speakers, based on the likelihood of patterns in particular phonological environments. For second-/third-generation ethnically Chinese individuals, the overall rate of t/d deletion is higher than the other demographic groups. They consider this weak evidence for ethnolects, as the patterns do not persist across generations.

### 1.4 Research question

Returning to our focus, we ask: when linked cross-language segment categories differ in the degree and direction of phonetic variation for a given phonetic dimension, does the less variable category constrain or guide production of the equivalent category in a different language whose variation is skewed in the direction of the opposing language? This question hinges on there being an asymmetry in the shape of distributions for linked sounds, and is thus notably distinct from the notion of compactness (as formulated in Flege & Bohn, 2021; Kartushina & Frauenfelder, 2014), for which the compact shape in the L1 is mirrored by a compact shape in the L2. Thus adapting the framework from Harrington and Schiel (2017) and Harrington et al. (2018), we examine whether the structure of similar cross-language categories affects how crosslinguistic influence is manifested for a bilingual, and, ultimately, how pronunciation variants may be introduced to a community through its bilingual members. To assess whether phonetic variation in stop categories is susceptible to more transient cross-language effects, we consider the effects of proximity to a language switch (from Cantonese to English) and language mode.

We consider two variable patterns across the studies reported in this paper. These studies are united in that they focus on phonetic variation in stop categories in English, but have conceptually different sources of Cantonese *invariance*. We acknowledge that it is unfortunate that we can only address variation in English and not in Cantonese, but our abilities to examine variation in Cantonese are stymied by the lack of an appropriate, age- and style-matched Cantonese language corpus—that lacks English influence—with which we can compare early Cantonese–English bilinguals. We leave such a study to future work.^1^

**Figure 1.**
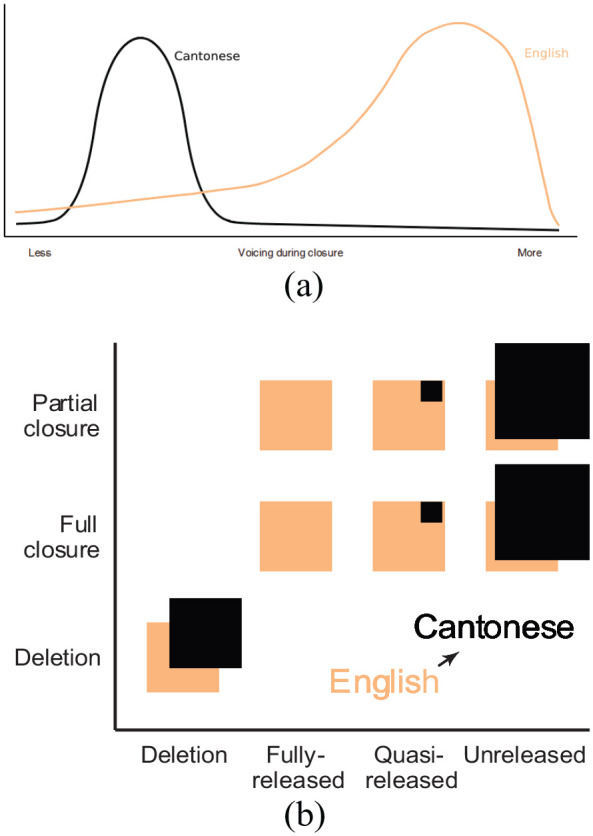
Schematizations of the distributions for (a) stop voicing during closure and (b) voiceless stop closure and release rates in word-final position. *Note.* In panel (a), the English distribution covers a much wider range of values compared with the Cantonese series. In panel (b), English is characterized by a wide range of variability for degree of both closure and release with boxes of the same size in each category, while Cantonese is assumed to primarily exhibit lenition-related variability and categorically lack a release. Given the highly variable nature of spontaneous speech, there would likely be occasional releases—as such, the distribution across discrete categories for Cantonese is depicted as heavily skewed. The squares in (b) are offset to ease visualization and do not suggest shifted patterns.

The first study focuses on sub-phonemic variation in the realization of voiced stops in English. The distribution of phonation during the closure for English /b d g/ is illustrated in Figure 1(a), where phonation is visualized here as a unitless less-to-more dimension. The English voiced stop series is highly variable in whether the voiced stop series truly shows phonation during the closure, ranging from voiceless unaspirated to containing phonation during the entire closure (Davidson, 2016; Fulop & Scott, 2021; Jacewicz et al., 2009). The distribution for phonation in English is skewed toward Cantonese. Conversely, the distribution for the equivalent series in Cantonese is more restricted. While the Cantonese stops /p t k/ are consistently described as voiceless unaspirated in single-word production with short-lag VOT (Lisker & Abramson, 1964; I. Y. H. Tsui & Ciocca, 2000), and are usually transcribed as /p t k/, the actual realization of these stops is more variable. Cantonese-acquiring children produce highly variable VOT, including many negative values, indicating phonation during the closure (Clumeck et al., 1981). In adults, Cantonese syllable fusion—a gradient prosodically conditioned reduction process—can lead to variable amounts of phonation in the unaspirated stop series as well (W. Y. P. Wong, 2006). Thus, while the descriptive literature on Cantonese states that these stops are produced in a voiceless unaspirated fashion (Bauer & Benedict, 2011), such descriptions may be more prescriptive than descriptive; hence, we illustrate phonation during these stops in Cantonese as a distribution centered around the less voicing end of the schematized unitless continuum, as opposed to a single discrete peak at no voicing end of the spectrum.

The second study examines the release behavior of voiceless final stops in English. Cantonese is described as categorically not releasing voiceless stops in syllable-final position, but oral occlusion is assumed (Bauer & Benedict, 2011; Matthews & Yip, 2013), while English release rates and degree of constriction are highly variable (Byrd, 1993; Davidson, 2011; Y. Kang, 2003). We expand on the discussion of final-stop variability in the introduction for Study 2. Final-stop patterns are modeled in Figure 1(b), in which the horizontal axis represents a selection of release behaviors—including deletion, unreleased, and released outcomes. The vertical dimension reflects categorical outcomes for the portion of the stop where closure occurs; in our schematization, stops may be deleted or exhibit a full or partial closure. A partial closure may be a stop that begins with frication and proceeds to a complete obstruction in the vocal tract. Neither the vertical nor the horizontal dimensions are to be interpreted as continuous, but are intended to show categories of patterns.^2^ The offset between Cantonese and English squares is to aid in visualization of the two patterns and is not intended to suggest shifts in the patterns. We model Cantonese /p t k/ as falling more heavily into the non-released outcome bins—though, given the nature of spontaneous speech, quasi-releases likely occur at least some portion of the time. This schematic glosses over the outcome of the presumed patterns in syllable fusion, which includes deletion and other forms of lenition. English, along both of these dimensions, is highly variable and skews toward the Cantonese patterns. Thus, our examination of this pattern probes how a more categorical phonological pattern from one language (Cantonese) may constrain a more variable pattern from another language (English). Further discussion of these phenomena is provided in the introductions to Study 1 and Study 2, respectively.

While the schematic distributions for phonation and word-final release rates for Cantonese differ in terms of their described categoricity or discreteness within Cantonese, they are both substantially less variable than English. Following the sound change models of Harrington and Schiel (2017) and Harrington et al. (2018), we predicted that the English of Cantonese–English bilinguals would be shifted toward the Cantonese distribution, as the more variable and skewed distribution encroaches onto the less variable distribution. That is, we predicted that English /b d g/ as produced by Cantonese–English bilinguals would have less voicing, and English /p t k/ in word-final position would have lower release rates as a result of mutual influence within the bilingual mind.^3^

## 2 Materials

The studies described in the paper make use of three spontaneous speech corpora. The first comprises Cantonese–English bilingual speech, and the latter two are monolingual control corpora.

### 2.1 Bilingual SpiCE corpus

The SpiCE corpus of bilingual speech in Cantonese and English includes high-quality laboratory recordings of conversational interviews with 34 early bilinguals in both languages (Johnson, 2021b), and is described in greater detail in Johnson (2021a) and Johnson et al. (2020).^4^ The Cantonese and English interviews were conducted by a pair of bilingual undergraduate research assistants, and lasted approximately 30 min each. The order of interviews was counterbalanced across participants within a two-interview session. SpiCE recordings are accompanied by orthographic, force-aligned transcripts in English and Cantonese.^5^

Half of the 34 speakers in the corpus reported their gender as female, and the other half as male. Speakers were generally university-aged (Mdn=21, M=22.4, SD=4), and learned both Cantonese (Mdn=0, M=0.03, SD=0.17) and English (Mdn=0, M=1.32, SD=1.77) from a young age. A total of 21 individuals report being simultaneous bilinguals, while the remaining 13 report sequential bilingualism. The sequential bilinguals all reported Cantonese as their first language, with English acquisition beginning between the ages of 2 and 5 years. Speakers reported high speaking proficiency in both English (Good/Excellent, n=33; Fair, n=1) and Cantonese (Good/Excellent, n=29; Fair, n=5). Self-ratings of Cantonese and English-speaking proficiency were equivalent for 16 speakers. In all, 6 speakers provided higher speaking proficiency ratings for Cantonese, and 12 gave a higher rating for their English. The linguistic profiles of the individuals in the SpiCE corpus are thus variable, but all are high-proficiency bilinguals.

Most speakers reported knowledge of Mandarin Chinese, and a variety of other languages—proficiency and age of acquisition for these other languages varied widely. For all speakers in the SpiCE corpus, English and Cantonese were among the earliest learned and highest proficiency languages. Most of the SpiCE bilinguals categorize their English variety as North American (n=16) ,^6^ Hong Kong English (n=4), or having influence from both varieties (n=9) . The rest report other varieties and combinations (e.g., British or Singaporean; n=5 ). The varieties of English are also reflected in where the speakers grew up. Most report being raised in Canada (n=11), Hong Kong (n=9), or a combination of the two (n=11). The remaining speakers grew up in Singapore (n=1), Canada and Singapore (n=1), or Hong Kong and Mainland China (n=1). The sample of early bilinguals in SpiCE is therefore heterogeneous, but is nevertheless representative of the Cantonese-speaking speech community in the region.

### 2.2 Monolingual comparison corpora

We compare SpiCE with two monolingual comparison corpora: a collection of sociolinguistic interviews with Vancouver, British Columbia (BC) English speakers (Swan, 2016), and the Buckeye Corpus of Conversational Speech (Pitt et al., 2005). Both collections comprise high-quality recordings of spontaneous monolingual English speech. These two comparison corpora were selected to give a comparison to a widely studied variety of English (Pitt et al., 2005), as well as the regional variety of English that many of the SpiCE bilinguals speak and all are exposed to (Swan, 2016). In both cases, it is not particularly important that the individuals are monolingual, per se, but rather that they (non-exhaustively) represent local and nonlocal English-speaking communities that do not have early multilingual experience with languages that would confound our line of inquiry.

The local spontaneous speech corpus collected by Swan (2016) includes sociolinguistic interviews with 40 speakers ranging in age from 18 to 36 years, and was recorded in 2014. We refer to this corpus as the Corpus of Transnational West Coast English (CTWCE). While this corpus also includes the speech of speakers from Seattle, WA, we only examined the speech of monolingual speakers from Vancouver (n=15) . Of this group, a majority were female (n=9), and the ages of the speakers were broadly similar to those in the SpiCE corpus (19–36 years of age). This corpus has been orthographically transcribed and force-aligned with the Montreal Forced Aligner (McAuliffe, Socolof, et al., 2017).

The Buckeye corpus is a collection of conversational interviews with 40 Caucasian monolingual English speakers from central Ohio, and data collection was completed in 2000 (Pitt et al., 2005). The interviews are transcribed and also include hand-corrected phonetic annotations. For this paper, we examine only the speech of the younger half of the Buckeye corpus (under <30; n=20). Half of the speakers were female, and half were male.

The inclusion of both these corpora allows us to be clear about what is a local English pattern that SpiCE and CTWCE share, versus what is a bilingual pattern that is not shared between SpiCE and either of the monolingual corpora. We note, however, that while these corpora all comprise spontaneous speech, they nonetheless have some key differences. Buckeye participants were interviewed by postdoctoral researchers and not told in advance that the purpose of the recording was for linguistic analysis. CTWCE, on the contrary, features sociolinguistic interviews recorded by Swan (2016), who was a graduate student at the time. In addition, these interviews included meta-linguistic discussion about dialect and language ideology. SpiCE is perhaps the most peer-to-peer of the three corpora, and while some aspects of the design were modeled after the Buckeye corpus, participants knew the purpose for recording (i.e., creating a bilingual corpus). In any case, all three corpora comprise conversational interview speech produced by younger adults, which renders them comparable.

## 3 General method

We consider two case studies where some degree of Cantonese influence is predicted to occur—intervocalic^7^ /b d g/ voicing (Study 1) and word-final /p t k/ release rates (Study 2). In both cases, we restrict the analysis to the set of environments in which we are *least* likely to see the predicted result in spontaneous speech, but where a difference between monolinguals and bilinguals is likely to be compelling, if present.

Using the transcripts from each corpus, we identified target instances of /b d g/ and /p t k/ using the PolyglotDB Python package (McAuliffe, Stengel-Eskin, et al., 2017), and compared the SpiCE bilinguals’ productions with each of the monolingual comparison corpora.^8^ The analyses described in the following studies report the results of mixed effects models for each study—cross-corpus comparisons, and a within-SpiCE analysis examining factors that influence language mode.

## 4 Study 1: Intervocalic /b d g/ voicing

The production of voicing for /b d g/ is highly variable within and across varieties of North American English, and across phonological environments within a variety or dialect (Davidson, 2016; Jacewicz et al., 2009). While these segments are less likely to contain voicing in phrase-initial position (Fulop & Scott, 2021; Keating, 1984), they are likely to exhibit full or partial voicing in other environments (Davidson, 2016; Fulop & Scott, 2021), with the proportion of voicing above 90% in intervocalic position for some dialects (Fulop & Scott, 2021; Jacewicz et al., 2009). Cantonese, on the contrary, lacks any phonemically voiced obstruents (Matthews & Yip, 2013). Bauer and Benedict (2011, p. 17) describe the initial stops in Cantonese as categorically voiceless and unaspirated, and suggest that American English speakers learning Cantonese “keep in mind the stop consonants of American English [b], [d], and [g] when they occur in word-initial position and refrain from voicing them.”

In this sense, English exhibits a wide range of acceptable variability, including production of /b d g/ without voicing, which parallels the voiceless unaspirated stop series in Cantonese. Given this disparity in variability, we predicted that bilinguals would produce less voicing during closure compared with monolinguals in English phrase-medial intervocalic productions of /b d g/—one of the environments in which voicing is most likely (Fulop & Scott, 2021). This follows from the parallels drawn with Harrington and Schiel (2017) and Harrington et al. (2018) in the “Introduction.”

### 4.1 Method

We identified all instances of phrase-medial intervocalic /b d g/, which includes stops in any word position, so long as they are phrase-medial. Manual inspection was used to identify gross errors and instances where the target segment was deleted.^9^ This is an important step, considering that we used force-aligned transcripts for two of the three corpora. In addition, by using the hand-corrected phonetic transcripts of the Buckeye corpus, instances of deletion in that corpus were not included in the initial sample. To this end, a single research assistant coded all target items as: (1) good, (2) deletion, or (3) transcript error. In addition, the research assistant indicated their confidence, identifying whether an item needed checking. Items that needed checking were coded by the first author (574 of 17,093). Prior to analysis, items were discarded if the original coder and author disagreed (1.4%), and if the coder labeled it confidently as a deletion (2.6%) or transcript error (1.9%). A total of 16,621 items were coded as “good” and used in the final analysis. Table 2 summarizes the counts used in the final set.

**Table 2.** Counts of /b d g/ Across the Three Corpora Included in the Final Analysis.

	/b/	/d/	/g/
SpiCE	1,736	2,958	1,344
CTWCE	549	757	270
Buckeye	2,656	4,451	1,900

*Note.* CTWCE = Corpus of Transnational West Coast English.

With this dataset, we measured the proportion of voiced frames between the onset and offset of the stop closure, using Praat’s *Voice Report* (Boersma & Weenink, 2020) implemented with the *Parselmouth* Python package (Jadoul et al., 2018). For both male and female talkers, the pitch floor was set to 50 Hz. The ceiling was set to 400 and 500 Hz, respectively.

### 4.2 Results

We analyzed the data using a generalized linear mixed effects model with a beta response distribution and logit link function. The model was implemented with the *glmmTMB* R package (Brooks et al., 2017). Beta regression is one of the recommended choices for modeling a proportional response variable that derives from a bounded continuous process (see discussion in Smithson & Verkuilen, 2006). In addition, the selection of the logit link function facilitates a log-odds interpretation analogous to logistic regression. The model formula used was: *Proportion Voiced* ~ *Corpus × Place × Duration* + *Position* + *Stress Precedes* + *Stress Follows* + *Word Frequency* + *(1 | Word)* + *(1* + *Place* + *Duration | Talker)*.^10^ The variables in the model were calculated and coded as indicated below. Many of the variables were implemented with weighted effect coding, which facilitates the interpretation of effects and interactions when the levels are not balanced, as is often the case with corpus data (Nieuwenhuis et al., 2017). In weighted effect coding, levels are compared against a weighted mean for that variable.

Proportion voiced is a continuous value ranging between 0 and 1, and represents the proportion of voiced frames in the target (i.e., voicing during closure). While for any given instance the proportion is based on a count of total frames, the underlying process is continuous. In order to facilitate beta regression, values were “squeezed” in the analysis, such that $y\prime \prime =\frac{y^{\prime }(N-)1+0.5}{N}$ , ensuring there were no 1’s and 0’s in the analysis. This is the dependent variable.

Corpus is a categorical fixed effect variable identifying the corpus an instance came from. Corpus was simple-coded with three levels—SpiCE, CTWCE, and Buckeye—with SpiCE as the reference level. This ensures that the comparisons of interest were made (SpiCE vs. CTWCE and SpiCE vs. Buckeye), and that the contrasts sum to 0, which facilitates the interpretation of variables involved in interactions. Corpus is the primary parameter of interest in this study.

Place is a categorical fixed effect variable identifying the place of articulation of the stop that was measured with levels B, D, and G. Place was weighted effect coded, with G as the reference level [*Place B*: B = 1, D = 0, G = −1.41; *Place D*: B = 0, D = 1, G = −2.32]. Place (G) was selected as the reference level because we anticipated that the dorsal stop would behave most similarly across the corpora due to aerodynamic constraints on voicing at that place of articulation.

Duration of the target segment in seconds was centered and standardized—it is a continuous fixed effect variable. Duration is included as a control fixed effect, as the proportion of voicing is expected to decrease as duration increases, due to the aerodynamic constraints of maintaining voicing during obstruents.

Position is a binary fixed effect variable indicating whether the target occurred word-initially or not. Position was weighted effect coded [Initial = 1, Not Initial = −1.50]. Position was included as a control fixed effect, as initial stops were expected to surface with less voicing (Jacewicz et al., 2009).

Preceding Stress is a binary fixed effect variable indicating whether there was stress on the preceding vowel (primary or secondary stress were both included as stressed). Preceding Stress was weighted effect coded [True = 1, False = −1.94].

Following Stress is a binary fixed effect variable indicating whether there was stress on the following vowel (again, primary and secondary stress were both included as stressed). Following Stress was weighted effect coded [True = 1, False = −2.88].

Word frequency was tabulated, log-transformed, centered, and standardized from the corpora (as in Tanner et al., 2017). This was done on a within-speech community basis, such that SpiCE and CTWCE transcripts were considered together (both are local speech), and transcripts from both young and old Buckeye corpus talkers were used (nonlocal). This ensures that regional vocabulary is not under- or misrepresented. Word frequency is a continuous fixed effect variable. It was included as a control fixed effect, as frequency has been shown to interact with various lenition processes (e.g., Tanner et al., 2017).

Word indicates the word that a target occurred in. Note that full word forms are used. The model includes random intercepts for Word.

Talker indicates the individual who produced the target. The model includes random intercepts for Talker, with by-Talker random slopes for Place and Duration.

While it may be clear from the above that the analysis will center on the role of Corpus—accounting for Place—we include a variety of other parameters to control for prosodic and probabilistic aspects that are known to impact a wide variety of processes in spontaneous speech. This decision follows from prior corpus-based work, such as work by Tanner and colleagues on the topics of coronal deletion (Tanner et al., 2017) and stop voicing during closure (Tanner et al., 2020). We discuss the expected outcomes of these variables in the context of reporting our results and the interim discussion.

The beta regression model returned with a significant intercept [β=1.85] indicating that items in the SpiCE corpus have a high proportion of voicing when all other variables are at their zero point—the weighted mean. Recall that the model uses a logit link function, and that results are interpreted on a log-odds scale. All of the model’s fixed effects are reported in Table 3, and we describe the significant results in text (at a significance level of p<.05), reporting parameter estimates. Refer to Table 3 for *SE*, *t*, and *p* values.

**Table 3.** Generalized Linear Mixed Effects Model Output for the /b d g/ Comparison Across Corpora in Study 1.

Parameter	Estimate	*SE*	*t*	*p*
Intercept	1.85	0.04	41.17	<.001
Corpus (CTWCE)	0.10	0.10	0.92	.36
Corpus (Buckeye)	0.25	0.09	2.74	.006
Place (B)	0.16	0.03	5.92	<.001
Place (D)	−0.10	0.02	−5.73	<.001
Duration	−0.56	0.03	−21.72	<.001
Position (initial)	−0.10	0.01	−8.61	<.001
Stress precedes (True)	0.03	0.01	3.73	<.001
Stress follows (True)	−0.04	0.01	−5.49	<.001
Word frequency	0.04	0.01	3.10	.002
Corpus (CTWCE) × Place (B)	0.13	0.06	2.09	.04
Corpus (Buckeye) × Place (B)	0.10	0.05	2.22	.03
Corpus (CTWCE) × Place (D)	−0.11	0.04	−2.77	.006
Corpus (Buckeye) × Place (D)	−0.05	0.03	−1.66	.10
Corpus (CTWCE) × Duration	0.09	0.06	1.32	.19
Corpus (Buckeye) × Duration	0.18	0.05	3.49	<.001
Place (B) × Duration	0.13	0.02	6.28	<.001
Place (D) × Duration	0.01	0.01	1.00	.32
Corpus (CTWCE) × Place (B) × Duration	−0.01	0.06	−0.22	.82
Corpus (Buckeye) × Place (B) × Duration	0.003	0.03	0.08	.94
Corpus (CTWCE) × Place (D) × Duration	0.004	0.04	0.11	.91
Corpus (Buckeye) × Place (D) × Duration	−0.02	0.02	−1.00	.32

*Note.* Estimates are to be interpreted as log-odds, given the beta distributed dependent variable and logit link function. CTWCE = Corpus of Transnational West Coast English.

**Figure 2.**
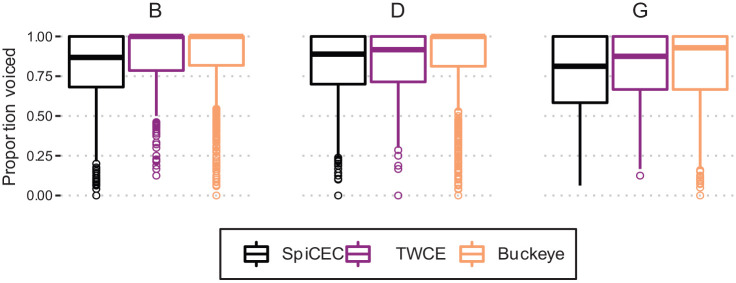
Proportion voiced for /b d g/ for the three corpora.

There were significant differences between the SpiCE corpus and both monolingual corpora, though the difference emerged in different ways. The estimate for the comparison between SpiCE and Buckeye [β=.25] indicates that Buckeye items were more likely to have a higher proportion of voicing. This was particularly true for Place (B), both across the board [β=.16], and specifically in the Place (B) × Corpus (Buckeye) interaction [β=.10]. This pattern is apparent in Figure 2, which shows clear differences in proportion voiced at all places of articulation for the SpiCE–Buckeye comparison.

**Figure 3.**
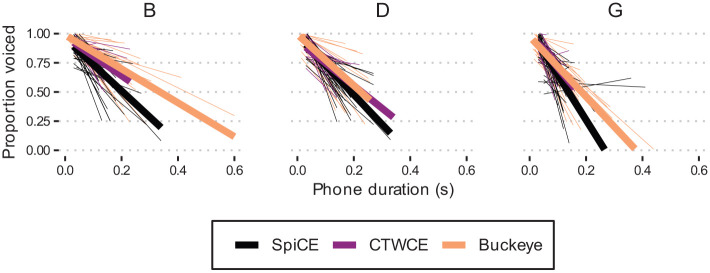
Proportion voiced by duration for /b d g/ for the three corpora. *Note.* Lines are linear smooths. Each thin line is for a single participant, and the thicker lines represent group means.

While the effect of Duration indicates that longer stops likely have a lower proportion of voicing [β=−.56] this effect was attenuated in the case of the Buckeye corpus [β=.18], and for items with Place (B) more generally [β=.13]. The effects of duration are depicted in Figure 3, with shallower group mean linear smooths for Buckeye stops compared with SpiCE across the board. Together, these results suggest that the Buckeye and labial stops exhibit higher proportions of voicing more generally—a conclusion that is corroborated by Figures 2 and 3.

In the case of the CTWCE corpus, the differences with SpiCE surface only in the interactions, such that Place (B) in the CTWCE corpus is more likely to have a higher proportion of voicing compared with SpiCE [β=.13]. Place (D) is overall likely to have a lower proportion of voicing compared with the weighted mean [β=−.10]. In addition, the Place (D) × Corpus (CTWCE) interaction [β=−.11] and the simple effect for Place (D) indicate that—as suggested by the empirical results in Figure 2—SpiCE and CTWCE do not differ substantially in the proportion of /d/ voicing. Figure 2 facilitates the interpretation of the Place (D) interaction, and shows a general pattern of CTWCE falling in between SpiCE and Buckeye.

As all of the categorical fixed effect variable contrasts sum to 0, it is possible to get reasonable main effect estimates from the model (Nieuwenhuis et al., 2017). In light of this, more frequent words were slightly more likely to be voiced [β=.04]. The proportion of voicing was also affected by prosodic environment, as anticipated. Stops in initial position were less likely to be voiced [β=−.10], and /b d g/ items which preceded a stressed vowel were less likely to be voiced [β=−.04], while post-tonic /b d g/ are more likely to be voiced [β=.03].

### 4.3 SpiCE-only /b d g/ model

A second generalized linear mixed effects model focuses on just the target items occurring in the SpiCE corpus. As before, beta regression with a logit link function was used to model the proportion of voicing during closure. The goal of this model was to understand how language mode impacts the proportion of voicing for /b d g/. Language mode is captured here in the variables described below—Code-switch Distance and Interview Order. The *glmmTMB* formula was: *Proportion Voiced ~ Place* × *Duration* × *Code-switch Distance* *+* *Interview Order* *+* *Position* *+* *Stress Precedes* *+* *Stress Follows* *+* *Word Frequency* *+* *(1 | Word)* *+* *(1* *+* *Place* *+* *Duration | Talker)*.^11^ Variables were defined in the same way as the 3-corpus model, with the exception of two new fixed effect variables:

Code-switch Distance is a continuous variable that captures proximity to a preceding code-switch into Cantonese. It was measured in seconds, and subsequently standardized. Prior work with spontaneous speech finds that crosslinguistic influence in code-switching is anticipatory, rather than perseverative (Fricke et al., 2016), though both directions of influence have been found in the experimental settings (Bullock & Toribio, 2009). For instances of /b d g/ that did not have a preceding switch, we used the onset of the English recording session, which then includes the elicitation of English sentences and the narration of an English story. We acknowledge that the onset of the English recording session is not a perfect proxy for the nearest code switch, as we have no knowledge of when the speaker would have last spoken Cantonese either as a code-switch or not.

Interview Order is a binary variable indicating whether or not the Cantonese interview preceded the English interview in the SpiCE recording session.^12^ Interview Order was weighted effect coded [Cantonese First = 1, English First = −1.03].

In addition, weights were recomputed for all other categorical variables with weighted effect coding—Place [*Place B*: B = 1, D = 0, G = −1.29; *Place D*: B = 0, D = 1, G = −2.20], Position [Initial = 1, Not Initial = −1.53], Following Stress [True = 1, False = −2.81], and Preceding Stress [True = 1, False = −2.06].

The model intercept was significant [β=1.74], indicating that stops in the SpiCE corpus were likely to have a high proportion of voicing. For the parameters that this model shares with the 3-corpus model, the output is largely the same, and detailed model output is reported in Table 4. This includes significant effects for Place (B) [β=.09], Place (D) [β=−.05], Duration [β=−.62], and Place (B) × Duration [β=.12]. There were also significant effects of Position (Initial) [β=−.14], Word frequency [β=.08], and Stress [Precedes: β=.03, Follows: β=−.06]. These effects can be interpreted in the same fashion as for the 3-corpus model reported in the previous section.

**Table 4.** Generalized Linear Mixed Effects Model Output for the [b d g] Comparison Within the SpiCE Corpus in Study 1.

Parameter	Estimate	*SE*	*t*	*p*
Intercept	1.74	0.05	32.25	<.001
Code-switch distance	−0.02	0.02	−1.17	.24
Place (B)	0.09	0.04	2.14	.03
Place (D)	−0.05	0.03	−1.83	.07
Duration	−0.62	0.03	−17.88	<.001
Interview order (Cantonese first)	−0.09	0.05	−1.88	.06
Position (initial)	−0.14	0.02	−7.19	<.001
Stress precedes (True)	0.03	0.01	2.43	.02
Stress follows (True)	−0.06	0.01	−5.08	<.001
Word Frequency	0.08	0.03	3.08	.002
Code-switch distance × Place (B)	−0.02	0.03	−0.93	.35
Code-switch distance × Place (D)	0.03	0.02	1.74	.08
Code-switch distance × Duration	0.005	0.02	0.25	.80
Place (B) × Duration	0.12	0.03	4.49	<.001
Place (D) × Duration	0.03	0.02	1.59	.11
Code-switch distance × Place (B) × Duration	0.02	0.02	0.62	.53
Code-switch distance × Place (D) × Duration	−0.01	0.02	−0.73	.46

**Figure 4.**
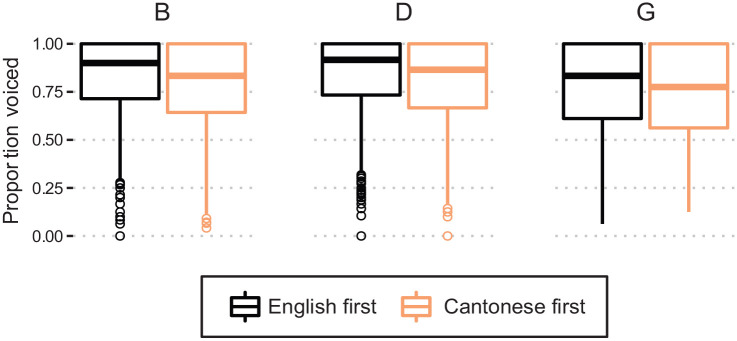
Proportion voicing during closure for /b d g/ within the SpiCE corpus, by interview order.

**Figure 5.**
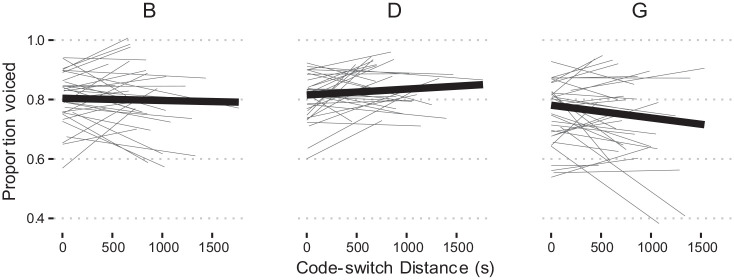
Proportion voicing during closure by code-switch distance for /b d g/ in the SpiCE corpus. *Note.* Lines are linear smooths, where thin lines represent participants, and thicker lines represent group means by Place.

There was no significant effect of Interview Order in the model, despite the consistent direction depicted in Figure 4. It is worth noting that this fixed effect was close to the threshold for significance [β=−.09, p=.06], but also that there was considerable between-subject variability. In addition, there was no significant effect of Code-switch Distance in the SpiCE corpus, an outcome that is not unexpected given the variation depicted in Figure 5. This is likely due to the relatively long distance from any given token to the nearest code-switch [n=372, Mdn=345.7s]. The previous literature documenting proximity effects typically considers a window well under 10 s (e.g., Fricke et al., 2016).

### 4.4 Interim discussion: /b d g/

These results largely follow predictions that individuals in the SpiCE corpus voice phonologically voiced stops less than individuals in the monolingual English corpora, which we interpret as evidence for Cantonese influence on the English spoken by these early Cantonese–English bilinguals. Recall that the selected phonological environment is where we expect the *most* voicing—a voiced stop immediately preceded and followed by vowels in peer-to-peer conversational speech. The evidence that early Cantonese–English bilinguals showed less voicing than monolinguals in phonologically voiced stops was found in a context that favors voicing (i.e., in intervocalic voiced stops). Thus, it is possible that this difference between these two groups in the voicing of stops may be even greater in contexts in which there is no coarticulatory pressure for voicing to occur in stops.

While we observe what we might call a global effect of Cantonese, we did not find a significant effect of language mode, as Interview Order narrowly missed the significance threshold. It may be possible that intervocalic voicing patterns are not sensitive to language mode, even if the bilingual individuals had entered into a particular language mode during the interview. Given the high degree of variability across individuals, we posit that the effects of Interview Order may warrant individual-specific exploration at a future point. While the effect of Interview Order might be considered marginal, there was no effect of Code-switch Distance (Figure 5). Target /b d g/ items that are closer to a Cantonese switch were not less likely to be voiced, as shown by the flat line, summarizing the group behavior. There was a long time lag between target /b d g/ and switches, as switches were infrequent in English interviews for most of the SpiCE talkers. Thus, this lack of effect is not surprising. Figure 5 also shows individuals’ mean lines, which are highly variable, suggesting an investigation of individual differences may prove fruitful.

The finding that stops in initial position were less likely to be voiced replicates the finding based on read sentences from Jacewicz et al. (2009) that voiced stops in prominent positions or subject to emphasis are less likely to be voiced in spontaneous speech. Like in Davidson (2016), stops that followed a stressed vowel were more likely to be voiced than those that followed an unstressed vowel. Stops that preceded an unstressed vowel were more likely to be voiced than stops that preceded a stressed vowel.

## 5 Study 2: word-final /p t k/ release rates

Final consonant deletion and lenition—particularly for coronal stops—has been widely studied within the sociolinguistics literature. Within English, coronal stop deletion and release rates are affected by style and dialect differences, in addition to phonological and morphological conditioning factors (e.g., Guy, 1980; Labov, 1989; Tagliamonte & Temple, 2005; Tanner et al., 2017). Word-final release rates for /p t k/ vary drastically in English across varieties and positions, with reported release rates ranging from 20% to 75% in pre-pausal, pre-nasal, and pre-obstruent positions (Davidson, 2011). Prevocalic positions are cited as a context that encourages stops to release and discourages deletion (see Davidson, 2011). As a result, most researchers examine stop behavior in consonant environments. Studies of the highly heterogeneous TIMIT corpus (Garofolo et al., 1993) confirm that release rates in American English are quite varied with estimates, which differ based on the corpus sample and exclusionary criteria, ranging from 52% unreleased for sentence-final postvocalic stops (Y. Kang, 2003) to 40% unreleased overall for sentence-final stops (Byrd, 1993). In Byrd’s analysis, stop release rates vary considerably by place of articulation: bilabial stops were released 50% of the time, alveolar stops 57% of the time, and velar stops 83% of the time (Byrd, 1993). Together, this suggests that final-stop behavior is highly variable in North American English.

Cantonese has voiceless /p t k/ in syllable-final position. These stops are described as categorically unreleased in coda position (Bauer & Benedict, 2011; Matthews & Yip, 2013), which is often considered to be a neutralization of the long-lag (aspirated) and short-lag (unaspirated) stops found in syllable-initial position (see Table 1). Because of this, Cantonese-speaking listeners rely heavily on information in the adjacent vowel to identify the place of articulation of final stop consonants (Khouw & Ciocca, 2006).

In this study, we examine prevocalic and pre-approximant word-final stops, as these environments appear to encourage stop releases. The goal is to test whether early Cantonese–English bilinguals show a lower incidence of stop release for word-final /p t k/ in English, compared with monolingual English speakers of two different varieties (local and nonlocal). Here, English has a wide range of acceptable variability, while Cantonese exhibits relative invariance for a similar set of consonants. In this case, crosslinguistic influence comes from a categorical phonological process in Cantonese. This source of influence differs markedly from the more phonetic and gradient influence found with /b d g/ voicing, as Cantonese stops are categorically unreleased in codas. While the laryngeal gesture for final stops is ambiguous, Matthews and Yip (2013) describe the process as a neutralization of short- and long-lag stop VOT in final position. Given this description, we predicted that bilinguals would be less likely to release English word-final stops compared with monolinguals.

It is important to highlight that this prediction is situated within the context of spontaneous speech between peers from the same speech community, and that we are predicting a difference in the extent to which different outcomes occur, not the absence of a pattern. Polinsky (2018) reports on an unpublished 2007 study involving a small sample of Cantonese–English heritage bilinguals, which found that heritage speakers were more likely to release final stops in English than a monolingual age-matched group. While this finding runs counter to our predictions, there are some crucial differences in task and communicative context. Polinsky indicates that releasing stops is used as a clear speech strategy, and may have been “amplified by the nature of the task as the subjects [were] asked to read the English material in a formal test setting” (Polinsky 2018, p. 144). In contrast, SpiCE participants were in conversation with a bilingual peer—a communicative context in which clear speech is less often necessary. The hyperarticulation for Cantonese–English heritage speakers reported in Polinsky (2018) may be a clear speech strategy that aligns with the characterization of bilinguals as sociolinguistically sophisticated in their capacity to use a wide variety of forms (Bullock & Toribio, 2009). Indeed, given Chang’s (2016) finding that early experience with Korean—another language with unreleased final stops—facilitates the perception of unreleased stops, Cantonese–English bilinguals may be particularly adept at deploying variation in a way that suites the stylistic and social demands of the context.

### 5.1 Method

All instances of word-final /p t k/ that preceded a vowel or approximant were identified. The segment preceding the target instance was not restricted, though vowels were by far the most frequent. Two research assistants coded all items by inspecting a waveform and spectrogram (dynamic range set to 65 dB; intensity contour superimposed), and by listening. Items were coded as one of six categories (adapted from Davidson, 2011). These categories were as follows:

(1) Release indicates that stop closure was followed by an abrupt, transitory aperiodic event, regardless of whether or not there was aspiration.

(2) Unreleased indicates that there was a clear stop closure, but no release.

(3) Lenition indicates there was no stop closure, but rather frication or formant structure in place of the stop. For example, this includes instances like “Eat an apple,” where /t/ flaps.

(4) Glottalization is characterized by irregular glottal striations in the preceding segment (if it was a vowel), and throughout the stop closure. While glottalization may accompany other categories, glottalization was only selected if there was evidence of glottal tension throughout.

(5) Deletion was used for cases with no stop closure or evidence of lenition. Research assistants were instructed to default to lenition in cases in which they were unsure.

(6) Transcript error indicates that the transcript was wrong in some way. This could be due to substantial misalignment, or selection of the wrong pronunciation variant (e.g., “w” transcribed as “double-u” rather than as a phonological fragment [w]). In most cases, this resulted from forced alignment errors.

Inter-rater reliability (IRR) between the two coders was assessed using Fleiss’s Kappa. While there was substantial agreement between the two raters for the six categories ( κ=.61 , p<.01 ), instances where there was disagreement were coded by a third research assistant (n=3,938) . As the primary point of interest in this study is whether or not a word-final stop releases, we collapsed lenition and glottalization into the unreleased category for the purposes of discarding observations. In practical terms, if rater A selected glottalization and rater B selected unreleased for the same item, this was treated as agreement.

From the initial total of 13,630, observations were then discarded if the three raters disagreed (1.9%), or if the consensus was transcript error (2.2%) or deletion (15.9%). Coders identified 163 deletions for Buckeye, 340 for CTWCE, and 1,668 for SpiCE. The comparatively smaller number of deletions for Buckeye is expected, as we used the surface transcriptions, which means that many instances of deletion were not included in the initial sample. While stop deletion is an interesting phenomenon, it is not the focus of this study, and we do not analyze it further. Table 5 summarizes the counts of each stop by corpus used in the analysis, from a total of 10,906 items. Table 5 also provides counts for the analysis described in the following section, which focuses specifically on the unreleased category, and uses a slightly smaller subset. While counts for all segments are provided in Table 5, we opted to exclude all /p/ items from the analysis, given the small and potentially non-representative sample—particularly in the case of CTWCE. After removing /p/ items, there was a total of 10,215 items.

**Table 5.** Counts of /p t k/ Across the Three Corpora Used in the Analysis.

	/p/	/t/	/k/
SpiCE	259 (251)	3,341 (3101)	2,494 (2,397)
CTWCE	53 (49)	859 (789)	444 (409)
Buckeye	379 (374)	1,114 (1079)	1,963 (1,898)

*Note.* The value in parentheses is the subset used for a more fine-grained unreleased analysis described in the following section. Note that while counts for /p/ are noted here, they were excluded from the analyses. CTWCE = Corpus of Transnational West Coast English.

### 5.2 Results

We analyzed the data with a logistic mixed effects model using the *lme4* R package (Bates et al., 2015). The *glmer* formula used was: *Released True ~ Corpus × Place × Rate* *+* *Vowel Precedes* *+* *Stress Precedes* *+* *Stress Follows* *+* *Word Frequency* *+* *(1 | Word)* *+* *(1* *+* *Place × Rate | Talker)*. Unless a difference is reported below, these variables were computed and coded as in Study 1. The new variables were as follows.

Rate is the average segment duration in the word. It was calculated as the word duration divided by the number of segments in the canonical representation of the word, and is intended as a proxy for speech rate. We opted to use a within-word measure, as some of the words in question are phrase-initial, and preceding context could not be used. This continuous fixed effect variable was centered and standardized.

Place is a binary fixed effect variable that accounts for place of articulation. Place was weighted effect coded [T = 1, K = −1.08].

Vowel Precedes is a binary fixed effect variable that indicates whether the preceding segment was a vowel or not. While the coronal stop deletion literature focusing on varieties of North American English often includes a more fine-grained categorization schema (e.g., Hazen, 2011), we used a simplified comparison that better matches Cantonese phonology (i.e., given that Cantonese does not have consonant clusters, the distinction is better assessed as vowels, which are licit in Cantonese, or not vowels, which are illicit in Cantonese). Vowel Precedes was weighted effect coded [True = 1, False = −3.48].

As in Study 1, Corpus was simple coded with SpiCE as the reference level, and weighted effect coding was used for Preceding Stress [True = 1; False = −10.65] and Following Stress [True = 1; False = −4.03].

**Figure 6.**
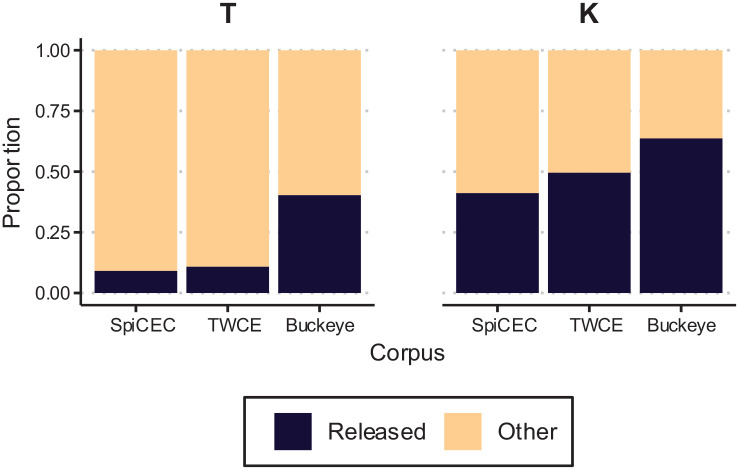
The proportion of released /t k/ items by corpus. *Note.* Refer to Table 5 for the counts by corpus. Other includes lenition, glottalization, and unreleased outcomes.

The logistic model returned a significant intercept [β=−1.47] , indicating that released stops were the less likely outcome across the board. All model output is reported in Table 6. Parameter estimates are reported in the text when significant (at a level of p<.05 ). There was an effect of the Buckeye Corpus [β=1.33], but not CTWCE. Both SpiCE and CTWCE had /t k/ release rates of 23% and 24%, respectively, whereas Buckeye had a 55% release rate. Figure 6 depicts the proportion of Released (True) for Place by Corpus.

**Table 6.** Logistic Mixed-Effects Model Comparing Release Rates for /t k/ Across the Three Corpora in Study 2.

Parameter	Estimate	*SE*	*z*	*p*
Intercept	−1.47	0.20	−7.49	<.001
Corpus (CTWCE)	0.31	0.22	1.44	.15
Corpus (Buckeye)	1.33	0.18	7.23	<.001
Place (T)	−1.29	0.12	−10.70	<.001
Rate	0.70	0.06	11.78	<.001
Vowel precedes (True)	−0.47	0.04	−11.23	<.001
Stress precedes (True)	0.003	0.01	0.19	.85
Stress follows (True)	−0.11	0.01	−7.87	<.001
Word frequency	−0.43	0.10	−4.45	<.001
Corpus (CTWCE) × Place (T)	0.07	0.18	0.40	.69
Corpus (Buckeye) × Place (T)	0.24	0.15	1.63	.10
Corpus (CTWCE) × Rate	−0.42	0.14	−3.02	.003
Corpus (Buckeye) × Rate	−0.35	0.12	−2.99	.003
Place (T) × Rate	−0.11	0.06	−2.00	.045
Corpus (CTWCE) × Place (T) × Rate	0.16	0.14	1.15	.25
Corpus (Buckeye) × Place (T) × Rate	0.17	0.11	1.45	.15

*Note.* CTWCE = Corpus of Transnational West Coast English.

**Figure 7.**
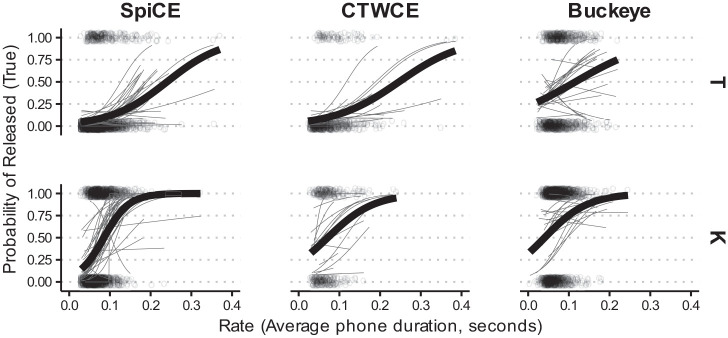
Probability of released (True) by place and rate. *Note.* On the *y*-axis, 1 corresponds to released, and 0 to not released (which includes the various forms of non-release). Individual items are plotted with translucent circles. The lines are generalized linear smooths (logit), where thick lines correspond to group means and thin lines to individuals.

There was an effect of Place (T) [β=−1.29] , indicating that /t/ was less likely to be released, but Place did not interact with Corpus. There was an effect of Rate [β=.70] —slower speech rates were accompanied by more released stops. In addition, Rate interacted with both Corpus (CTWCE) [β=−.42] and Corpus (Buckeye) [β=−.35], which indicates that the differences in release rates compared with SpiCE are diminished as average phone duration within the word increases. Rate interacted with Place (T) [β=−.11], which indicates that increased chance of release at slower speech rates is attenuated for coronals. These results are depicted in Figure 7.

More frequent words were less likely to be released [β=−.43]. When /t k/ followed a vowel, the stop was less likely to be released [β=−.47] than if that stop was preceded by a consonant. If the word-final /t k/ was followed by a vowel that is stressed, that stop is less likely to be released [β=−.11].

### 5.3 Unreleased /t k/ model

The above analysis establishes that SpiCE bilinguals and CTWCE monolinguals release stops at similar levels. While the analysis mirrors the structure of prior work on final stops, it does not take account of the greater likelihood for Cantonese–English bilinguals to produce final stops as *unreleased*. In light of this, we present an additional more exploratory analysis. We flip the question and model the incidence of unreleased final stops specifically—clear stop closure, and *no* burst—as opposed to all other phonetic variants. While it may seem counterintuitive to lump glottalization and lenition in with released final stops, this approach addresses the question of how likely stops are to be produced as they are in Cantonese—as *unreleased* stops.

The model was nearly identical to the model of the previous section, with the exception that the dependent variable here is Unreleased, a binary variable with values True and False. In addition, as this model is based on the more fine-grained coding scheme, we examine the subset of the dataset in which two of the three raters agreed on the unreleased category—totaling 9,673 of the initial 10,215 instances of /t k/. Counts by place of articulation and corpus are reported in parentheses in Table 5. In addition, weighted effect coding was updated for Place [T = 1, K = −1.06], Vowel Precedes [True = 1, False = −3.52], Preceding Stress [True = 1, False = −11.06], and Following Stress [True = 1, False = −4.03]. The model formula was: *Unreleased True ~ Corpus × Place × Rate* *+* *Vowel Precedes* *+* *Stress Precedes* *+* *Stress Follows* *+* *Word Frequency* *+* *(1 | Word)* *+* *(1* *+* *Place × Rate | Talker)*.

**Figure 8.**
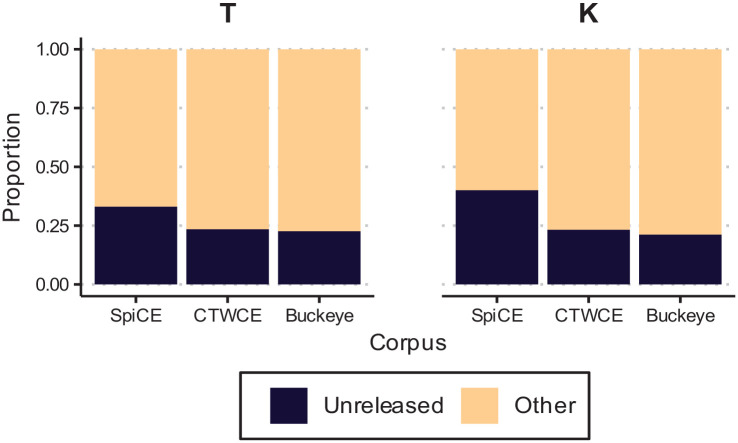
The proportion of tokens that are unreleased by place and corpus. *Note.* In this plot, “Other” includes released, glottalization, and lenition.

**Figure 9.**
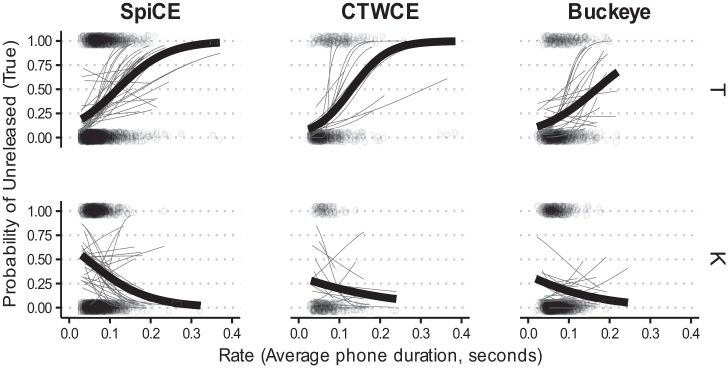
Probability of being unreleased by rate. *Note.* The value 1 corresponds to unreleased, and 0 to *not* unreleased (which includes released, lenition, and glottalization). Individual items are plotted with translucent circles. The lines are generalized linear smooths (logit), where thick lines capture group means, and thin lines correspond to individuals.

The model results are provided in Table 7. The intercept was significant [β=−1.05], indicating that unreleased stops were less likely than the alternative surface forms in aggregate. SpiCE items were more likely to be unreleased, compared with both monolingual corpora [Buckeye: β=−.62, CTWCE: β=−.51] —a pattern that is clearly depicted in Figure 8. There was also a significant Corpus (Buckeye) *×* Place (T) interaction [β=.33], suggesting that the comparison between SpiCE and Buckeye differs across the two places of articulation. Rate [β=.25] interacted with Place (T) [β=.50], which suggests that at slower speech rates, /t/ is more likely to be unreleased. This is shown in Figure 9, and we offer a hypothesis for this pattern below.

**Table 7.** Logistic Mixed-Effects Model Output for the /t k/ Comparison for the More Fine-Grained Coding of Unreleased.

Parameter	Estimate	*SE*	*z*	*p*
Intercept	−1.05	0.20	−5.38	<.001
Corpus (CTWCE)	−0.62	0.19	−3.30	<.001
Corpus (Buckeye)	−0.51	0.16	−3.15	.002
Place (T)	0.25	0.11	2.20	.03
Rate	0.25	0.06	4.41	<.001
Vowel precedes (True)	0.20	0.04	4.93	<.001
Stress precedes (True)	−0.04	0.01	−3.61	<.001
Stress follows (True)	0.07	0.01	5.36	<.001
Word frequency	0.31	0.10	3.03	.002
Corpus (CTWCE)× Place (T)	0.15	0.14	1.01	.31
Corpus (Buckeye) × Place (T)	0.33	0.12	2.72	.006
Corpus (CTWCE) × Rate	0.20	0.13	1.48	.14
Corpus (Buckeye) × Rate	−0.02	0.11	−0.18	.85
Place (T) × Rate	0.50	0.05	10.00	<.001
Corpus (CTWCE) × Place (T) × Rate	0.001	0.12	0.01	.99
Corpus (Buckeye) × Place (T) × Rate	−0.15	0.10	−1.50	.13

*Note.* CTWCE = Corpus of Transnational West Coast English.

For the control fixed effects, the presence of a preceding vowel [β=.20] led to an increased probability of being unreleased. The probability of being unreleased also was affected by Stress [Precedes: β=−.04, Follows: β=.37], and Word Frequency [β=.31]. That is, a stop was less likely to be unreleased when it occurred before a stressed syllable, and more likely to be unreleased when it followed a stressed syllable. More frequent words were more likely to be unreleased.

### 5.4 SpiCE-only /t k/

For the same reasons as in Study 1, we examined the role of language mode on /t k/ production in a separate analysis focusing on just the SpiCE corpus. As differences between SpiCE and the local monolingual comparison group (CTWCE) were evident in the unreleased model, we adapted that model to probe effects of language mode. As such, the dependent variable reflects whether or not a particular item was coded as unreleased in the fine-grained coding scheme. The additional variables in the SpiCE-only model were coded in the same fashion as Study 1, with the weighted effect coding updated for Place [T = 1, K = −1.29], Interview Order [Cantonese First = 1, English First = −1.06], Vowel Precedes [True = 1, False = −5.24], Stress Precedes [True = 1, False = −9.82], and Stress Follows [True = 1, False = −4.37]. The *glmer* formula was: *Unreleased True ~ Place × Rate × Code-switch Distance* *+* *Interview Order* *+* *Vowel Precedes* *+* *Stress Precedes* *+* *Stress Follows* *+* *Word Frequency* *+* *(1 | Word)* *+* *(1* *+* *Place × Rate | Talker)*.

Model output is summarized in Table 8. The model returned a significant intercept [β=−.87], indicating that unreleased was a less likely outcome than released, lenition, and glottalization considered together.

**Table 8.** Logistic Mixed-Effects Output Modeling the Probability of /t k/ Items Being Unreleased Within the SpiCE Corpus in Study 2.

Parameter	Estimate	*SE*	*z*	*p*
Intercept	−0.87	0.22	−3.94	<.001
Place (T)	0.19	0.11	1.72	.09
Rate	0.22	0.06	3.67	<.001
Code-switch distance	0.07	0.04	1.79	.07
Interview order (Cantonese first)	0.07	0.09	0.76	.45
Vowel precedes (True)	0.05	0.03	1.61	.11
Stress precedes (True)	−0.05	0.02	−3.02	.003
Stress follows (True)	0.07	0.02	4.57	<.001
Word frequency	0.10	0.11	0.93	.35
Place (T) ×Rate	0.48	0.05	8.77	<.001
Place (T) × Code-switch Distance	−0.05	0.03	−1.47	.14
Rate × Code-switch Distance	−0.04	0.04	−1.15	.25
Place (T) × Rate × Code-switch Distance	0.04	0.03	1.13	.26

**Figure 10.**
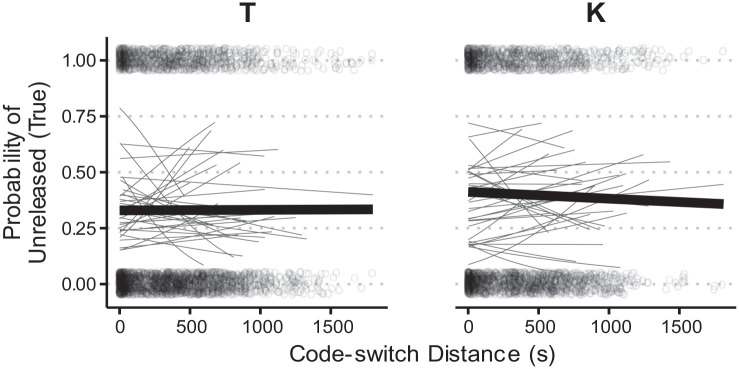
The probability of an item being unreleased is plotted against code-switch distance. *Note.* Translucent circles represent individual tokens. The thin lines represent individuals, and thick lines represent group means. Despite the flat appearance of the group means, the lines are all generalized linear smooths (logit).

The primary variables of interest in this model—Code-switch Distance and Interview Order—exerted no significant effect on the response variable and did not participate in any interactions. If Code-switch Distance were to be considered given its marginal status [β=.07, p=.07], it would run counter to the hypothesis, as a positive effect suggests that stops are more likely to be unreleased as distance from a Code-switch increases. Given the extremely flat smooths for group means and high degree of individual variability in Figure 10, it seems more plausible that this is a spurious marginal result. As with the /b d g/ SpiCE-only model, instances of /t k/ were generally quite far from a code-switch (Mdn=335.7s), and the nonsignificant result is thus not particularly surprising.

The control variables in the model largely mirrored the 3-corpus model in effect size and significance, and can be interpreted in the same fashion. There were significant effects for Rate [β=.22] and a Rate × Place (T) interaction [β=.48], though the simple effect of Place (T) missed the significance threshold [β=−.19, p=.09]. There were also significant effects for Stress [Precedes: β=−.04, Follows: β=.07]. The effect of preceding vowel missed the cutoff for significance [β=.05, p=.11], but trends in the same direction as the 3-corpus model. Unlike the 3-corpus model, there was no effect of Word Frequency on the probability of an item being unreleased. This could be due to any number of reasons, all of which would be highly speculative in nature.

### 5.5 Interim discussion: /t k/

The results for /t k/ ultimately follow the prediction that English release patterns would be influenced by Cantonese. This does not surface for release rates, but rather for *unreleased* rates. There are two primary factors contributing to this outcome. First, release rates were surprisingly low in the local monolingual speech from CTWCE (24%) and comparable to the release rates for SpiCE (23%), compared with the more widely studied nonlocal variety in the Buckeye corpus (55%). This highlights that our expectations about what the local variety would do were not entirely correct. While prior work on BC English has not examined stop release rates, there are a variety of documented features unique to the area (e.g., Mellesmoen, 2018), and the fact that CTWCE and Buckeye do not show the same pattern further contributes to this understanding of BC English. Second, talkers in the SpiCE corpus are more likely to produce unreleased /t k/ compared with both groups of monolinguals—local and nonlocal. Following from descriptions of Cantonese final stops, this outcome is indicative of crosslinguistic influence, as the bilinguals are producing word-final English stops with Cantonese-like settings the most frequently of the three groups.

While these models provide evidence for crosslinguistic influence, it is ultimately a coarse picture, as the nature of the releases and non-releases is under-described in the literature. For example, while we find that SpiCE talkers are more likely to have unreleased /t k/, this makes no comment about the nature of their released tokens, which include both full releases and quasi-releases. In addition, there is also the question of what happens in Cantonese, and whether quasi-releases can occur in spontaneous speech. These categorical results build on prior work (e.g., Davidson, 2011), and invite future research in this area.

There was no effect of Interview Order or Code-switch Distance. While language mode is widely studied, it is possible that the nature of final releases is more codified as a feature of the bilinguals’ English and thus less subject to these more ephemeral forms of interference. It is also possible that our decision to categorize items as released and unreleased obscures the effect of online crosslinguistic influence. This would be the case if the degree of release were to pattern gradiently, like proportion voicing for /b d g/. Again, these results raise questions that invite future research, which would additionally involve describing whether and how quasi-releases surface in Cantonese final stops.

In addition to the main findings for /t k/, we also report on the unique behavior of /k/ across all three corpora. At slower rates /k/ was the most likely to release, which might be due to the unique behavior of frequent words within this class such as *like* (Drager, 2011). Indeed, “like” accounts for 73% of /k/ items in the SpiCE corpus, 56% in CTWCE, and 50% in the Buckeye corpus. The unique behavior of /k/ could also hinge on standard phonetic mechanisms, as the increase in inter-oral pressure behind a velar constriction may essentially require a release more than stops at more anterior places of articulation (Byrd, 1993; Cho & Ladefoged, 1999). This is also evident in how Place patterns in the models—/t/ is less likely to be released. /t/ releases pattern in the same way as /k/ with respect to speech rate, with the probability of /t/ being released increasing at slower speech rates. In the case of the unreleased model, /t/ is more likely to be unreleased, even as speech rate slows down (unlike /k/). The increased unreleased rates in this environment likely reflect an increased prevalence of lenition at faster speech rates for /t/.

## 6 General discussion

We examined pronunciation differences in English /b d g/ and /t k/ as spoken by early Cantonese–English bilinguals (SpiCE corpus: Johnson, 2021b) compared with a local English monolingual control group (CTWCE: Swan, 2016) and a well-studied English monolingual control group from a different North American English speech community (Buckeye corpus: Pitt et al., 2005). The SpiCE corpus consists of spontaneous speech from 34 early Cantonese–English bilinguals, who have high speaking proficiency in both languages. Cantonese speakers worldwide are a multilingual group, making this particular sample representative of the larger Cantonese speech community.

A goal of this work was to bring together the specific predictions from recent theories on sound change (Harrington et al., 2018; Harrington & Schiel, 2017) with the well-established empirical findings that a bilingual’s languages exhibit mutual influence (Best & Tyler, 2007; Flege & Bohn, 2021; Kroll & Navarro-Torres, 2018; Simonet, 2016), which credit the existence of mutual influence to parallel activation and connections between linguistic units, but do not offer, to our knowledge, a concrete mechanistic account of asymmetry in crosslinguistic influence for similar, linked segments in early bilingual speech production. There are, of course, a variety of mechanisms that have been proposed to explain why speech sounds should influence each other in the first place in L2 speech perception and production (e.g., Best & Tyler, 2007; Flege & Bohn, 2021). The mechanism proposed here deepens our understanding of what happens within what Flege and Bohn’s (2021) SLM-r would term as a composite category. SLM-r can account for why a pair of sounds may be linked through notions of similarity, and our approach helps account for what happens within such a linked composite category. Note that we are not implying that a composite category is a unimodal merging of two cross linguistic categories, though in late L2 learners, that may certainly be the case.

In describing what makes this mechanism unique, we touch on some points made in the “Introduction.” While considerations of category compactness may be important for the learning outcomes of late L2 learners (Kartushina et al., 2015; Kartushina & Frauenfelder, 2014; Kartushina, Frauenfelder, & Golestani, 2016; Kartushina, Hervais-Adelman, et al., 2016), category compactness may be less important for phonetic category formation and cross linguistic mutual influence for early bilinguals (Werker & Byers-Heinlein, 2008). In addition, Olson (2016) and Bullock and Toribio (2009) observe that some sound categories may have more “room for influence” or phonetic space, inviting variability and providing opportunity for bilinguals to exhibit sociolinguistic sophistication of the stylistic deployment of particular linguistic features. This concept of room for influence combined with the work by Harrington and colleagues invites us to ask whether the distribution of phonetic variation in linked sound categories across a bilingual’s languages goads pronunciation variation in the direction of the less variable language when the more variable language skews that way.

In the two studies we explored here, Cantonese was the less variable language and the variation in English was skewed in the direction of Cantonese. Study 1 examined the proportion of voicing during the stop closure for English /b d g/. As presented in Table 1, the phonologically linked sounds in Cantonese are the word-initial voiceless unaspirated stops /p t k/, which, while described in Cantonese grammars as voiceless unaspirated, are also described as being similar to the English voiced stop series (Bauer & Benedict, 2011; Matthews & Yip, 2013), in addition to showing phonation in acquisition (Clumeck et al., 1981) and in morpho-phonological processes (W. Y. P. Wong, 2006). Thus, while there is some indication that phonation for Cantonese /p t k/ is gradient, it is much less variable than what is permitted in English for /b d g/, which skews into the Cantonese distribution. We predicted, therefore, that Cantonese–English bilinguals would show effects of the less variable language (Cantonese) pulling on the skewed distribution of the more variable language (English). Indeed, we found that Cantonese–English bilinguals were less likely to produce phonation during /b d g/ than either of the monolingual English control groups. We interpret this as an indication that the English in early Cantonese–English bilinguals shows effects of influence from Cantonese. In /b d g/ voicing patterns we also looked at the role of language mode. While SpiCE bilinguals who completed a Cantonese interview before the English interview showed a pattern of producing less voicing compared with those who completed the English interview first, the effect was marginal. Thus, while voicing may be susceptible to language mode—either operationalized in a more sensitive way or as part of a different task—we do not establish evidence for it here. While our results are consistent with Harrington and colleagues’ mechanism, Lein et al. (2016) provide VOT data from a small sample of French–German simultaneous bilinguals that may counter predictions about crosslinguistic influence being driven by the structure of variance. More research focused on the mechanisms of *why* asymmetries crop up in crosslinguistic influence is certainly necessary.

The phonological pattern of /p t k/ in word-final position in Cantonese is different. Obstruent codes in Cantonese are categorically described as unreleased (Bauer & Benedict, 2011; Khouw & Ciocca, 2006; Matthews & Yip, 2013), though susceptible to lenition in the context of syllable fusion (W. Y. P. Wong, 2006). Cantonese word-final /p t k/ is much less variable than Cantonese word-initial /p t k/ realizations than in English word-final stops. Thus, while we therefore predicted that, again, the less variable pattern (Cantonese) will pull the pattern of the skewed and more variable language (English) toward it, the more categorical nature of the absence of final-stop releases in Cantonese makes this pattern conceptually quite different.

We report initial counts for each of the three segments, but opted to only analyze /t/ and /k/, as there were relatively few instances of /p/ across the three corpora, which raised concerns about the representativeness of the sample. The initial analysis modeling the likelihood of word-final English /t k/ being released established a difference between SpiCE and the nonlocal monolingual variety (Buckeye), but not between SpiCE and the local CTWCE corpus. Rather, we uncovered a rather surprising regional difference, as both Cantonese–English bilingual and monolingual Vancouver speech patterns had very low rates of stop releases in the word-final position. We consider instead the likelihood that stops occurred with a closure and were unreleased (and thus grouping other realizations together: lenition, glottalization, and full closure with release). In this analysis, we found that SpiCE talkers were more likely to produce unreleased stops than either CTWCE or Buckeye talkers. The unreleased rates of word-final English /t k/ showed no effects of language mode or (interpretable) Code-switch Distance, suggesting that the pattern uncovered in the unreleased model is a codified feature of Cantonese–English bilingual speech and not subject to more transient effects—at least, as coarsely implemented here. Alternatively, if release and non-release patterns are actually gradient, then it is possible the analysis here is not sensitive enough to detect differences in release patterns based on language mode.

A finding separate from the core questions about bilingual speech that merits attention is the unique differences between /t k/ in word-final unreleased rates. At slower speech rates, the probability of /t/ for all three corpora being unreleased increased; at slower speech rates, unreleased /t/ was more likely. The pattern for /k/, however, is the opposite and robust: for all three corpora, the likelihood of /k/ being unreleased decreases as speech rate slows; at slower speech rates /k/ is less likely to be unreleased. The pattern with /k/ appears convincing in Figure 9, but should also be accompanied with the caveat that a very high number of word-final /k/ are due to the word “like.” Whether the unique pattern for /k/ is due to peculiarities with this particularly multifaceted lexical item or due to post-constriction pressure increases that require a release (Cho & Ladefoged, 1999) is an issue we leave to future research.

In considering Cantonese, English, and their mutual influence, one must remember that languages in contact can take many tracks as a result of the social dynamics between speech communities and the linguistic structure of the languages (Thomason & Kaufman, 1988). A common language contact situation in contemporary Western societies is that of a diaspora speech community, where there is a shift to the societally dominant language, such that younger speakers might be characterized as heritage speakers of their ancestral language. While Polinsky and Scontras (2020) suggest there is little evidence for structural transfer as a result of contact-induced change in heritage languages, Muysken (2020) criticizes this jump to conclusions and cautions that such a characterization is likely due to a focus in the literature on bilingual immigrant communities that undergo rapid language shift in the highly monolingual and English-dominant context of the United States. Bilingual communities with longer histories, Muysken argues, are those more likely to show evidence of structural contact-induced change. The bilingual individuals whose speech is under study here are early bilinguals, who may or may not be more or less dominant in one language or another. Some—but not all—of the speakers in the SpiCE corpus may fall under the designation of heritage speakers in the sense that they were raised in an English-dominant society and primarily use Cantonese with family. As a whole, the Cantonese–English speech community in the Vancouver, BC area is well established, having existed since the latter half of the 19th century (Yu, 2013). Therefore, this speech community is a clear candidate for contact-induced change (Muysken, 2020).

In addition to considering the impacts of social dynamics at the level of the speech community (Thomason & Kaufman, 1988), it is also important to consider social and communicative factors within the speech sample being studied. At first glance, our hypotheses and results might appear incompatible with prior work demonstrating that bilinguals show patterns of divergence in the types of environments where we predict a distributional shift more similar to convergence. For example, Polinsky (2018) finds that Korean–English and Cantonese–English heritage bilinguals are more likely to produce word-final stop releases in English compared with monolinguals in an experimental setting. Following from Bullock and Toribio’s (2009) argument that bilinguals have a rich repertoire of forms at their disposal, it is perhaps unsurprising that bilinguals hyperarticulate in formal, experimental settings. Polinsky (2018, p. 144) speculates, however, “that heritage speakers’ tendency to release final stops . . . may be a side effect of a more general tendency to enunciate . . . [as] they are used to communicating in English with nonnative speakers who benefit from clear word boundaries.” Our results suggest a different explanation. If bilinguals are highly likely to produce released stops in a formal experimental setting, but are more likely to produce unreleased stops when conversing with a peer in their speech community, then an account of this behavior must take communicative context and interlocutors into account. As bilinguals are better at perceiving place of articulation in unreleased stops (Chang, 2016), it follows that unreleased stops are less likely to affect the intelligibility of conversations between Cantonese–English bilinguals. While this explanation remains somewhat speculative, our results offer yet another cautionary tale for the (implicit) assumption that findings from laboratory speech are replicated in spontaneous speech (e.g., see Gahl et al., 2012). This is particularly relevant in the case of the phenomena we study, as our focus is on how broad patterns shape the speech of communities. We identify this as a promising area for future work.

Altogether, this project suggests that the phonetic variation in crosslinguistically linked categories in bilingual speech is shaped by the distribution of phonetic variation within each language. This is a mechanistic, and not purely descriptive, account for why some segments are more susceptible to cross-language influence than others in studies of mutual influence. While we consider these results as evidence that the nature of within-category phonetic distributions can drive mutual influence across languages, we must, of course, acknowledge that we cannot make this claim strongly. The claim is tempered, as we only investigate the effect of Cantonese on English and not English on Cantonese, so we have no evidence of *mutual* influence. The multilingualism of Cantonese speakers, however, poses a challenge for fully addressing the research question with this particular set of languages, as we do not have access to a comparable spontaneous speech sample from Cantonese speakers whose linguistic knowledge is devoid of potential influence from another language that might confound the results—and in many cases, where the other language is specifically English. Our approach, however, brings new predictive rigor to the study of crosslinguistic influence and can be further tested, refuted, or refined in investigations of additional languages.

## Notes

1. While we do not offer the flipped perspective of English influence on Cantonese, we can offer some predictions. For the stop patterns investigated here, we predict limited influence of English on Cantonese because the variation in these stops skews English toward Cantonese and not Cantonese toward English. For any linked sounds where the direction of variation orients Cantonese toward English (and not the reverse), we would predict Cantonese to show more English influence. We leave room in the concept of mutual influence for more global influences due to mutual activation, whereas for the present purposes, we focus on more specific and targeted influence due to the distributional patterns.

2. The order of the axis labels may strike some as counterintuitive. The goal of the axes as presented is to facilitate the reading of these dimensions as categories of patterns and not a single gradient dimension.

3. As noted by a reviewer, our predictions may not be a shift toward Cantonese so much as a shift toward the typologically less-marked pattern. We do not address concepts like the emergence of the unmarked in this paper.

4. Documentation for the corpus can be found at https://spice-corpus.readthedocs.io/.

5. Cantonese transcription was primarily done in Cantonese characters, with Jyutping romanization restricted to cases for which there is no character.

6. North American here includes those who responded with “American” or “Canadian”—a more fine-grained self-reporting of English variety was not included.

7. As in much of the previous work using continuous speech, intervocalic here refers to any phrase-medial intervocalic stop whether the target sound be in word-initial, word-medial, or word-final position (e.g., Fulop & Scott, 2021; Jacewicz et al., 2009).

8. SpiCE and the sociolinguistic interviews from Swan (2016) were force-aligned using the Montreal Forced Aligner with the built-in English acoustic model and pronunciation dictionary (McAuliffe, Socolof, et al., 2017).

9. The Buckeye’s more narrow surface transcription was used, compared with the force-aligned transcripts for SpiCE and CTWCE. This means that what would likely be called a vowel-nasal sequence in SpiCE and CTWCE is a nasalized vowel in Buckeye and thus included in the analysis.

10. The model failed to converge with a Place × Duration interaction in the random effects structure.

11. The model failed to converge with a Place × Duration interaction in the random effects structure.

12. Note that while the order of interviews was counterbalanced across participants, counterbalancing did not extend to other demographic factors. For example, 10 of the 17 participants who completed the Cantonese interview first were female.

13. This differs from Davidson (2011) in that we lump all cases of quasi-release in with release.
